# Supplementary material for: A multiscale signalling network map of innate immune response in cancer reveals cell heterogeneity signatures
Source: Nat Commun. 2019 Oct 22;10:4808. doi: 10.1038/s41467-019-12270-x (PMC6805895; doi:10.1038/s41467-019-12270-x)
Supplement: Supplementary file 1 — Supplementary Information [file 41467_2019_12270_MOESM1_ESM.pdf]

SUPPLEMENTARY INFORMATION

Kondratova, et al.

A multiscale signalling network map of innate immune response in cancer reveals cell heterogeneity signatures

Complex CD14:IRAK2:IRAK4:LY96:MYD88:TIRAP:TLR2/4*

Complex composition:

1. CD14
2. IRAK4
3. TLR2/4*
4. LY96
5. MYD88
6. TIRAP
7. IRAK2

CD14:IRAK2:IRAK4:LY96:MYD88:TIRAP:TLR2/4*@INNATE_IMMUNE_CELL_Membrane

Identifiers
NAME:CD14:IRAK2:IRAK4:LY96:MYD88:TIRAP:TLR2/4*

Maps_Modules
macrophages_mdsc_cells
dendritic_cell
MODULE:IMMUNOSTIMULATORY_CYTOKINE_PATHWAYS
METAMODULE:IMMUNE_STIMULATION
LAYER:INDUCERS
ZONE:ANTI_TUMOR_POLARIZATION

SIGNALLING_PATHWAYS:TLR2_4

References
PMID:14660645, PMID:16878026, PMID:23681101
The adaptor proteins MyD88 and TIRAP associate with TLR 2 and TLR 4 after receptor engagement.
Transfection of dominant negative MyD88 or TIRAP inhibited almost all of the HMGB1- and LPS-induced NF-Î°B activation.
MyD88 recruits members of the death domain-containing serine/threonine IL-1R-associated kinase (IRAK) family.
PMID:12620219
MyD88 mediates a close interaction of the two related IRAK molecules,
which is essential to allow IRAK-4 to phosphorylate IRAK-1. Phosphorylation of IRAK-1
reduces its affinity for MyD88, while increasing its affinity for TRAF6
PMID:21372296
HMGB1 induces the production of angiogenic factors VEGF, and TGFB1 by macrophage via TLR4-dependent mechanisms.

Confidence
★★★☆☆ REF=3 FUNC=4

Supplementary Figure 1. Map entity annotation page in NaviCell format.

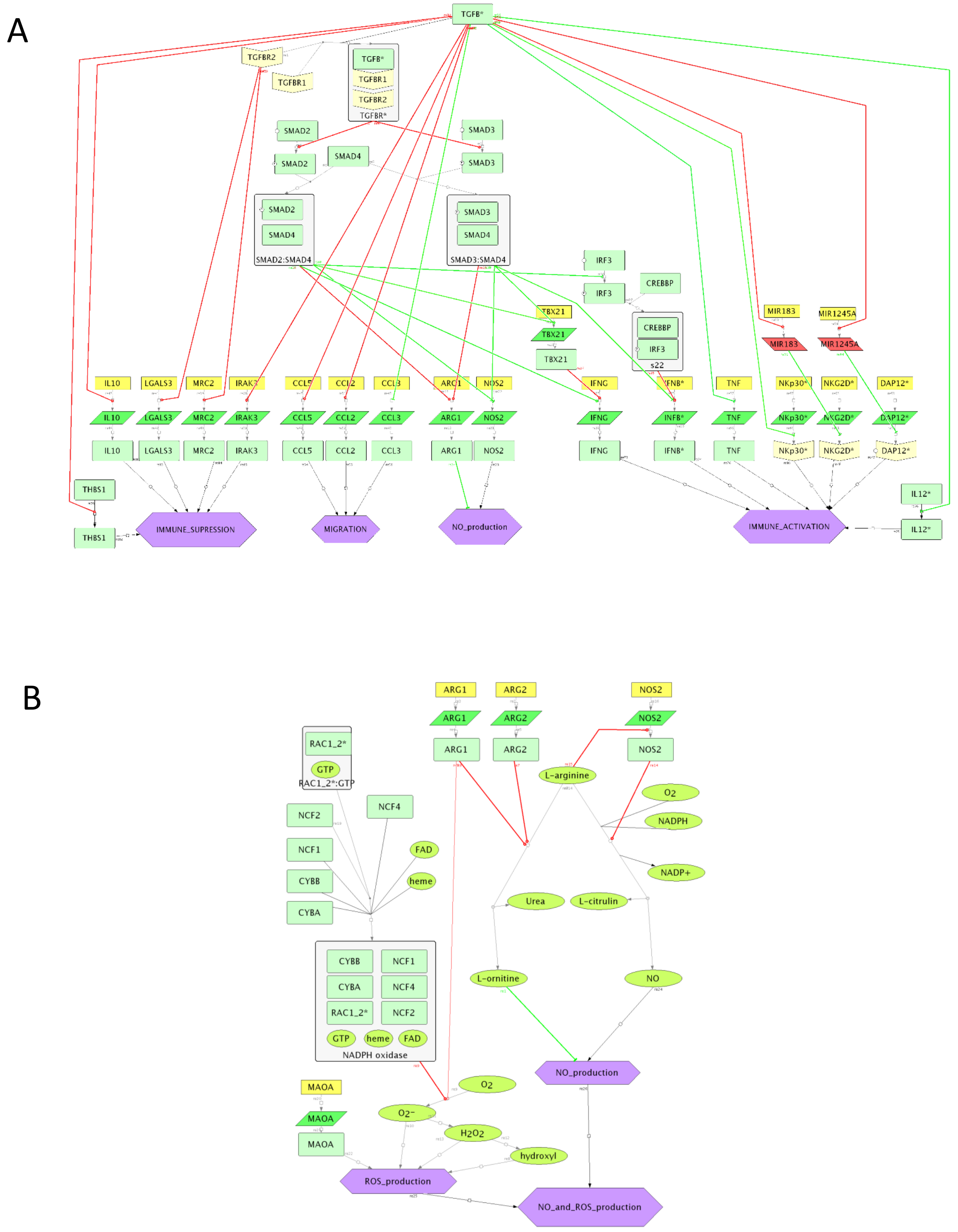

Supplementary Figure 2. Example of signalling pathways and functional modules extracted from the innate immune response in cancer meta-map by tags. (A) TGFB signalling pathway. (B) NO and ROS production functional module.

A

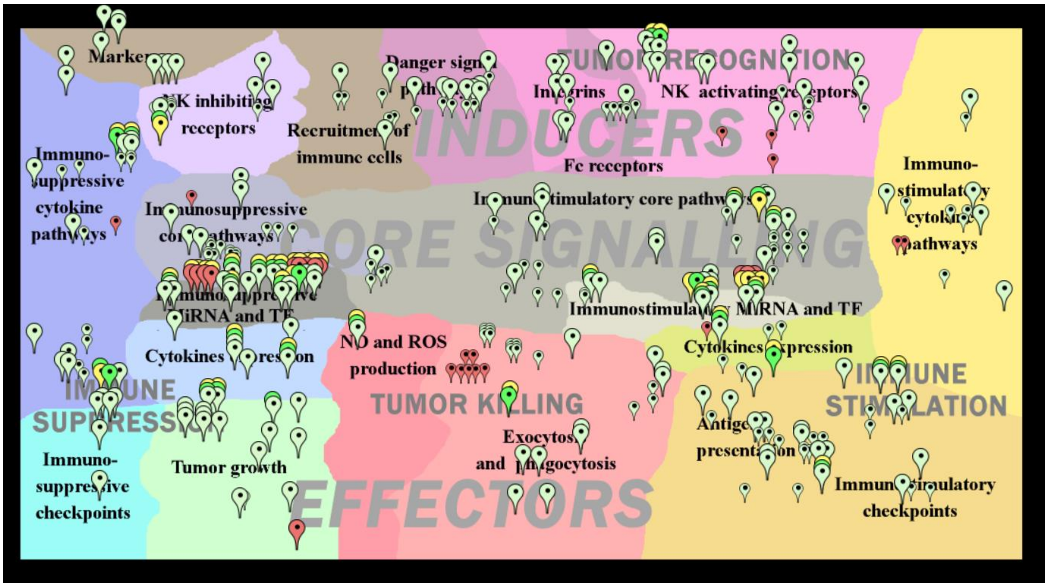

B

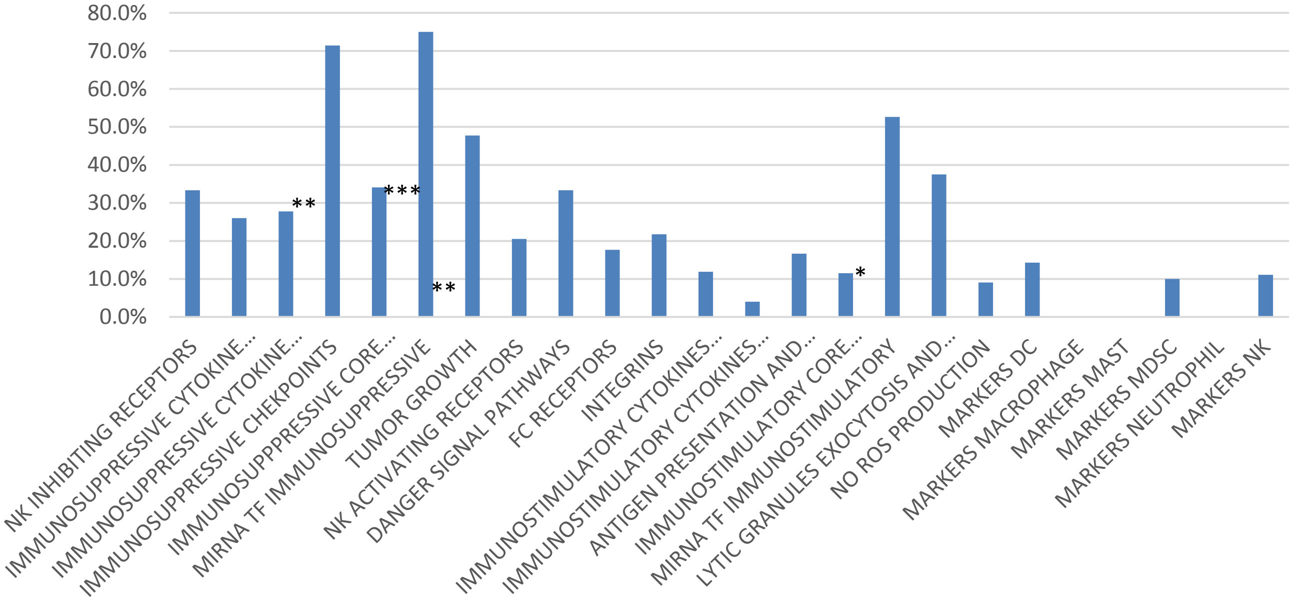

Supplementary Figure 3. Comparison of gene names content in InnateDB, REACTOME, KEGG databases and and in the innate immune response in cancer meta-map. (A) Visualization of 188 unique genes distribution from the meta-map across functional modules. The content of the map was compared with innate immune-related sub-set of pathways from Innate DB, KEGG and REACTOME and unique genes were identified and visualized. (B) Enrichment of functional modules on meta-map with the unique genes (percentage). The p-value of the χ-squared test is reported following the code: *** < 0.001, ** < 0.01, * < 0.05.

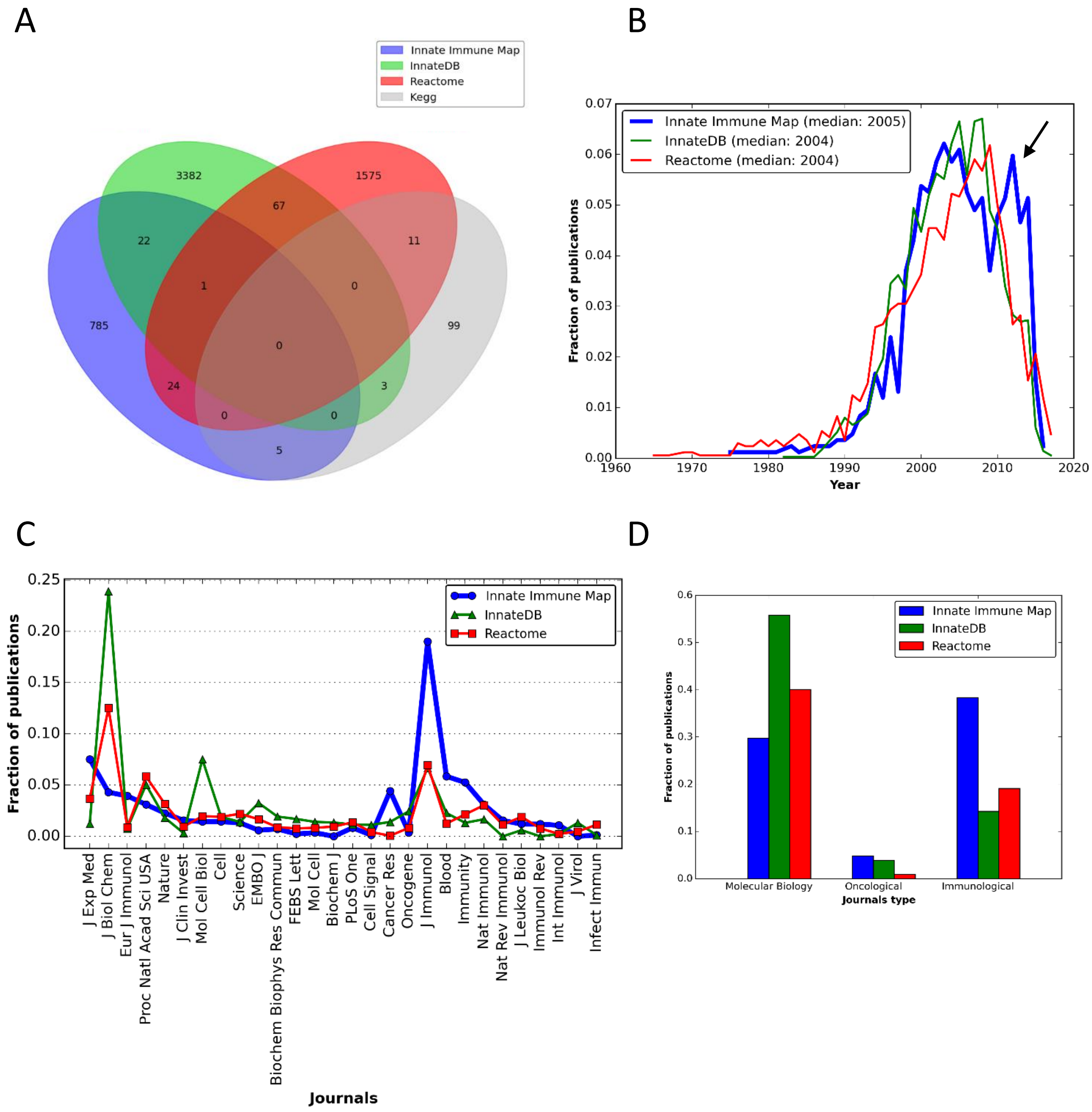

Supplementary Figure 4. Comparison of publications used for map annotation in InnateDB, REACTOME, KEGG databases and in the innate immune response in cancer meta-map. (A) Venn diagram showing intersection of the publications annotating the selected pathways from the three databases and the meta-map. There are 785 unique publications used in the meta-map. There are only several tenth of publications that are actually common between all databases, indicating uniqueness of each one of those. Distribution of (B) publication years and (C) journals annotating the selected pathways from InnateDB, REACTOME and the meta-map. The arrow indicates papers published during the period 2010-2015. (D) Relative distribution of journals types used for annotation of the selected pathways from InnateDB, REACTOME databases and of the meta-map.

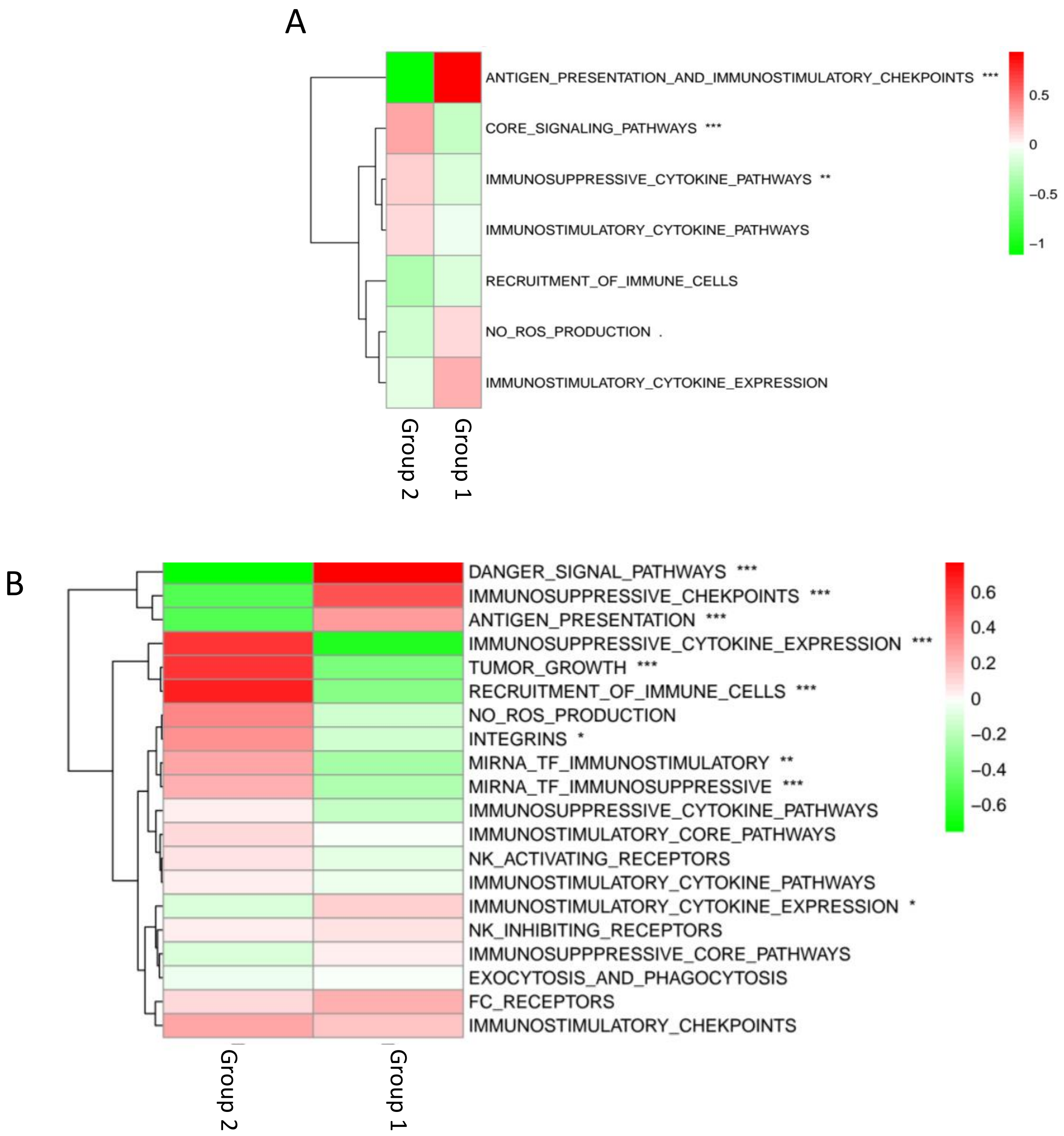

Supplementary Figure 5. **Macrophage cells sub-populations study.** Calculation of modules activity scores using expression data from melanoma macrophage cells and modules from innate immune response in cancer resource. Activity scores of Macrophages in the two groups for (A) cell type-specific map and for (B) meta-map. The p-value of the t-test between gene expression is reported following the code: *** < 0.001, ** < 0.01, * < 0.05, < 0.1

A

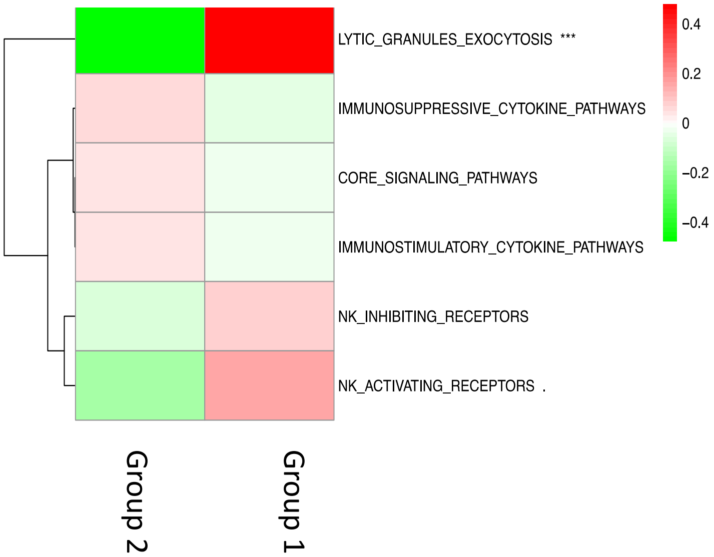

B

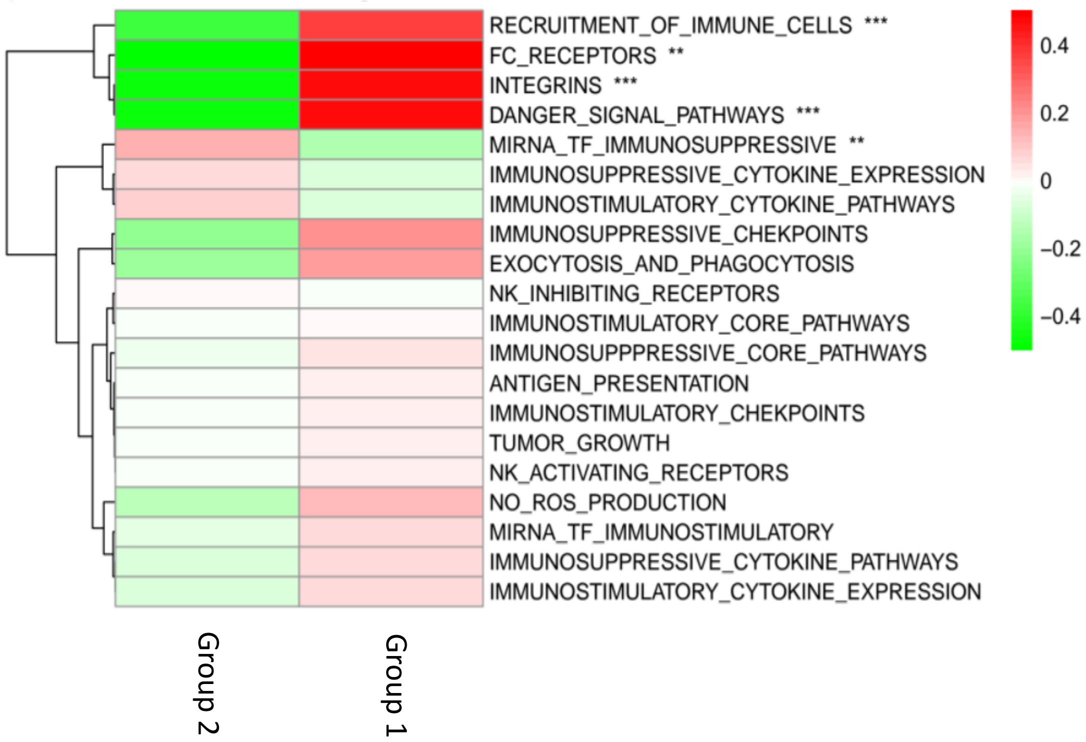

Supplementary Figure 6. Natural killers (NK) cells sub-populations study. Calculation of modules activity scores using expression data from melanoma (NK) cells and modules from innate immune response in cancer resource. Heatmap shows activity scores of each group in modules of (A) cell-type-specific map and (B) meta-map. The p-value of the t-test between gene expression is reported following the code: *** < 0.001, ** < 0.01, * < 0.05, < 0.1

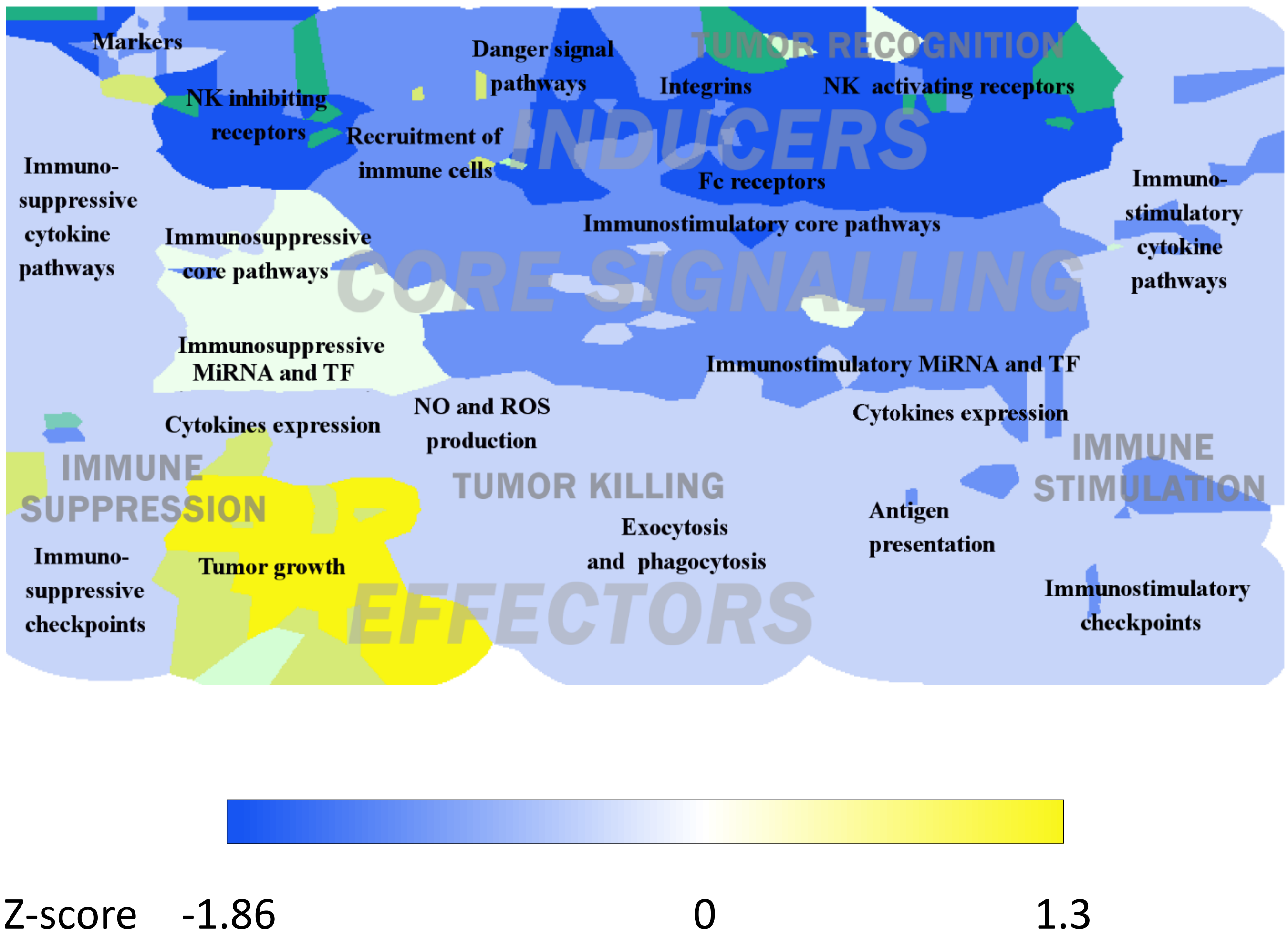

Figure 7. Innate immune response in cancer meta-map represents a potential source of patient's survival prognostic signatures. (A) Visualization of mean Z-scores of genes from meta-modules in the context of the meta-map. Blue zones are enriched with genes with a positive correlation to patient survival, yellow zones are enriched with genes correlated with negative patient survival.

Supplementary Table 1: Structure and content of cell-type specific innate immune maps

Map/Module	Chemical Species	Proteins	Genes	RNAs	asRNAs	Reactions	References
**Macrophages and MDSC**	**588**	**217**	**95**	**95**	**4**	**457**	**189**
RECRUITMENT OF IMMUNE CELLS	37	15	6	6	0	29	**18**
NO ROS PRODUCTION	54	20	6	6	0	37	**25**
IMMUNOSTIMULATORY CYTOKINE PATHWAYS	92	55	10	9	1	50	**75**
IMMUNOSTIMULATORY CYTOKINE EXPRESSION	81	31	20	21	0	76	**35**
ANTIGEN PRESENTATION AND IMMUNOSTIMULATORY CHEKPOINTS	16	5	5	5	0	15	**10**
CORE SIGNALLING PATHWAYS	144	58	21	21	1	102	**58**
IMMUNOSUPPRESSIVE CYTOKINE PATHWAYS	163	57	34	33	4	122	**82**
**Natural killers**	**567**	**249**	**53**	**42**	**14**	**377**	**309**
IMMUNOSTIMULATORY CYTOKINES PATHWAYS	107	46	18	15	5	81	**89**
CORE SIGNALLING PATHWAYS	125	71	5	6	0	140	**131**
IMMUNOSUPPRESSIVE CYTOKINE PATHWAYS	61	21	14	6	10	38	**48**
NK INHIBITING RECEPTORS	48	26	2	2	1	31	**72**
NK ACTIVATING RECEPTORS	124	66	7	8	3	63	**142**
LYTIC GRANULES EXOCYTOSIS	54	34	5	5	5	52	**45**
**Dendritic cells**	**491**	**226**	**43**	**44**	**1**	**346**	**278**
IMMUNOSTIMULATORY CYTOKINES PATHWAYS	132	66	20	21	0	89	**125**
ANTIGEN PRESENTATION	95	54	5	5	0	81	**67**
CORE SIGNALLING PATHWAYS	62	33	6	6	1	39	**31**
IMMUNOSUPPRESSIVE CHEKPOINTS	7	6	0	0	0	6	**12**
MARKERS DC	10	9	0	0	0	11	**12**
IMMUNOSUPPRESSIVE CYTOKINE PATHWAYS	58	28	8	8	0	37	**52**
RECRUITMENT OF IMMUNE CELLS	27	15	4	4	0	23	**11**
TUMOR RECOGNTITON TUMOR KILLING	54	26	2	2	0	37	**39**

Supplementary Table 2. Pathways from Innate DB, KEGG and REACTOME used for comparison with the innate immune response in cancer meta-map.

DATABASE/Pathways	DATABASE ID
INNATE DB	
Chemokine Signaling Pathway (Human)	
Cytosolic DNA-sensing Pathway (Human)	
Jak-STAT Signaling Pathway (Human)	
MAPK Signaling Pathway (Human)	
mTOR Signaling Pathway (Human)	
Natural killer cell mediated cytotoxicity (Human)	
KEGG	
Toll-like receptor signaling pathway	hsa04620
Cytosolic DNA-sensing pathway	hsa04623
Natural killer cell mediated cytotoxicity	hsa04650
	hsa04612
Antigen processing and presentation	hsa04664
Fc epsilon RI signaling pathway	hsa04666
Fc gamma R-mediated phagocytosis	hsa04062
Chemokine signaling pathway	hsa04670
Leukocyte transendothelial migration	
REACTOME	
Innate Immune System	R-HSA-168249
Interferon Signaling	R-HSA-913531.1
Signaling by Interleukins	R-HSA-449147.7
TNFR2 non-canonical NF-kB pathway	R-HSA-5668541.2
Class I MHC mediated antigen processing &	R-HSA-983169.3
presentation	R-HSA-2132295.3
MHC class II antigen presentation	
