# Supplementary material for: A multiscale signalling network map of innate immune response in cancer reveals cell heterogeneity signatures
Source: Nat Commun. 2019 Oct 22;10:4808. doi: 10.1038/s41467-019-12270-x (PMC6805895; doi:10.1038/s41467-019-12270-x)
Supplement: Supplementary file 3 — Description of Additional Supplementary Files [file 41467_2019_12270_MOESM3_ESM.pdf]

Description of Additional Supplementary Files

File Name: Supplementary Data 1
Description: List of genes from the innate immune response in cancer meta-map that positively or negatively correlate with the patient survival.

File Name: Supplementary Data 2
Description: Gene content of signalling pathways in innate immune response in cancer meta-map.

File Name: Supplementary Data 3
Description: Gene content of meta-modules and modules on innate immune response in cancer meta-map.

File Name: Supplementary Data 4
Description: Unique gene lists from the innate immune response in cancer meta-map in comparison to the selected pathways from InnateDB, KEGG and REACTOME databases.
