# Supplementary material for: A multiscale signalling network map of innate immune response in cancer reveals cell heterogeneity signatures
Source: Nat Commun. 2019 Oct 22;10:4808. doi: 10.1038/s41467-019-12270-x (PMC6805895; doi:10.1038/s41467-019-12270-x)
Supplement: Supplementary file 8 — Reporting Summary [file 41467_2019_12270_MOESM8_ESM.pdf]

natureresearch

Corresponding author(s): Inna Kuperstein

Last updated by author(s): 01 July 2019

Reporting Summary

Nature Research wishes to improve the reproducibility of the work that we publish. This form provides structure for consistency and transparency in reporting. For further information on Nature Research policies, see Authors & Referees and the Editorial Policy Checklist.

Statistics

For all statistical analyses, confirm that the following items are present in the figure legend, table legend, main text, or Methods section.

n/a	Confirmed	
☒	☐	The exact sample size (*n*) for each experimental group/condition, given as a discrete number and unit of measurement
☒	☐	A statement on whether measurements were taken from distinct samples or whether the same sample was measured repeatedly
☐	☒	The statistical test(s) used AND whether they are one- or two-sided<br>*Only common tests should be described solely by name; describe more complex techniques in the Methods section.*
☐	☒	A description of all covariates tested
☐	☒	A description of any assumptions or corrections, such as tests of normality and adjustment for multiple comparisons
☐	☒	A full description of the statistical parameters including central tendency (e.g. means) or other basic estimates (e.g. regression coefficient) AND variation (e.g. standard deviation) or associated estimates of uncertainty (e.g. confidence intervals)
☐	☒	For null hypothesis testing, the test statistic (e.g. *F*, *t*, *r*) with confidence intervals, effect sizes, degrees of freedom and *P* value noted<br>*Give P values as exact values whenever suitable.*
☒	☐	For Bayesian analysis, information on the choice of priors and Markov chain Monte Carlo settings
☐	☒	For hierarchical and complex designs, identification of the appropriate level for tests and full reporting of outcomes
☐	☒	Estimates of effect sizes (e.g. Cohen's *d*, Pearson's *r*), indicating how they were calculated

Our web collection on statistics for biologists contains articles on many of the points above.

Software and code

Policy information about availability of computer code

Data collection	GEO (GSE72056)
Data analysis	https://navicell.curie.fr/pages/maps_innateimmune.html<br>https://github.com/sysbio-curie/NaviCell/tree/master/auxiliary_scripts<br>https://github.com/sysbio-curie/NaviCell

For manuscripts utilizing custom algorithms or software that are central to the research but not yet described in published literature, software must be made available to editors/reviewers. We strongly encourage code deposition in a community repository (e.g. GitHub). See the Nature Research guidelines for submitting code & software for further information.

Data

Policy information about availability of data

All manuscripts must include a data availability statement. This statement should provide the following information, where applicable:

- Accession codes, unique identifiers, or web links for publicly available datasets
- A list of figures that have associated raw data
- A description of any restrictions on data availability

The cell type-specific maps and meta-map of innate immune response in cancer are freely available at the web page (https://navicell.curie.fr/pages/maps_innateimmune.html). The meta-map and cell type-specific maps are provided in three platforms, NaviCell, MINEVRA and integrated into the repository NDEx. The maps exist and can be downloaded in several exchange formats (CellDesigner SBML level 2 version 4, SBGN-ML 0.2, SBML level 3 version 1, Cytoscape CX version 3.4.0). In addition, the composition of map signalling pathways, modules and meta-modules is provided in a form of GMT files (Supplementary Tables 2 and 3 respectively) suitable for further functional data analysis. A network of binary relations between proteins generated from the meta-map and the complete list of references annotating the maps are also available. Finally, the documentation and the scripts for module activity calculation and generation of life example is provided at GitHub (https://github.com/sysbio-curie/NaviCell/tree/master/auxiliary_scripts). The step-by-step procedure on modular hierarchical maps construction is also provided at https://github.com/sysbio-curie/NaviCell.

Field-specific reporting

Please select the one below that is the best fit for your research. If you are not sure, read the appropriate sections before making your selection.

☒ Life sciences ☐ Behavioural & social sciences ☐ Ecological, evolutionary & environmental sciences

For a reference copy of the document with all sections, see nature.com/documents/nr-reporting-summary-flat.pdf

Life sciences study design

All studies must disclose on these points even when the disclosure is negative.

Sample size	NA
Data exclusions	NA
Replication	NA
Randomization	NA
Blinding	NA

Reporting for specific materials, systems and methods

We require information from authors about some types of materials, experimental systems and methods used in many studies. Here, indicate whether each material, system or method listed is relevant to your study. If you are not sure if a list item applies to your research, read the appropriate section before selecting a response.

Materials & experimental systems

n/a	Involved in the study
☒	☐ Antibodies
☒	☐ Eukaryotic cell lines
☒	☐ Palaeontology
☒	☐ Animals and other organisms
☒	☐ Human research participants
☒	☐ Clinical data

Methods

n/a	Involved in the study
☒	☐ ChIP-seq
☒	☐ Flow cytometry
☒	☐ MRI-based neuroimaging
